# Polyphenolic Composition and Antioxidant Activity (ORAC, EPR and Cellular) of Different Extracts of *Argylia radiata* Vitroplants and Natural Roots

**DOI:** 10.3390/molecules27030610

**Published:** 2022-01-18

**Authors:** Ady Giordano, Pablo Morales-Tapia, Mauricio Moncada-Basualto, Josué Pozo-Martínez, Claudio Olea-Azar, Aleksandra Nesic, Gustavo Cabrera-Barjas

**Affiliations:** 1 Inorganic Chemistry Department, Faculty of Chemistry and Pharmacy, Pontificia Universidad Católica de Chile, Avenida Vicuña Mackenna 4860, Santiago 8330077, Chile; agiordano@uc.cl; 2 Escuela de Ciencias Agrícolas y Veterinarias, Universidad Viña del Mar, Agua Santa 7055, Viña del Mar 2531015, Chile; pamorales1@uc.cl; 3 Laboratory of Free Radicals and Antioxidants, Faculty of Chemical and Pharmaceutical Sciences, University of Chile, Sergio Livingstone Polhammer 1007, Independencia 7820436, Chile; m.moncada.ba@gmail.com (M.M.-B.); jspozom1991@gmail.com (J.P.-M.); colea@uchile.cl (C.O.-A.); 4 Instituto de Ciencias Biomédicas, Facultad de Medicina, University of Chile, Santiago 8380453, Chile; 5 Unidad de Desarrollo Tecnológico (UDT), Universidad de Concepción, Avenida Cordillera 2634, Parque Industrial Coronel, Concepción 3349001, Chile; a.nesic@udt.cl; 6 Department of Chemical Dynamics and Permanent Education, Vinca Institute of Nuclear Sciences—National Institute of the Republic of Serbia, University of Belgrade, Mike Petrovica-Alasa 12-14, 11000 Belgrade, Serbia; 7 Centro Nacional de Excelencia Para la Industria de la Madera (CENAMAD), Pontificia Universidad Católica de Chile, Vicuña Mackena 4860, Santiago 7820436, Chile

**Keywords:** *Argylia radiata*, vitroplants, polyphenolic extracts, antioxidant activity, ORAC, electronic paramagnetic resonance (EPR)

## Abstract

Plant biochemistry studies have increased in recent years due to their potential to improve human health. *Argylia radiata* is an extremophile plant with an interesting polyphenolic profile. However, its biomass is scarce and occasionally available. *Argylia* in vitro biomass was obtained from tissue culture and compared with in vivo roots regarding its polyphenolic and flavonoid content. Different solvents were used to prepare extracts from the in vitro tissue of callus and aerial plant organs and in vivo roots. UPLC-MS/MS was used to assess the chemical composition of each extract. ORAC-FL and scavenging of free radicals (DPPH and OH) methods were used to determine the antioxidant capacity of extracts. Furthermore, the biological activity of the extracts was established using the cellular antioxidant activity method. The vitroplants were a good source of polyphenols (25–68 mg GAE/100 g tissue FW), and methanol was the most efficient solvent. Eight polyphenolic compounds were identified, and their antioxidant properties were investigated by different chemical methods with EPR demonstrating its specific scavenging activity against free radicals. All extracts showed cellular dose-dependent antioxidant activity. The methanolic extract of vitroplants showed the highest cellular antioxidant activity (44.6% and 51%) at 1 and 10 µg/mL of extract, respectively. Vitroplants of *A. radiata* are proposed as a biotechnological product as a source of antioxidant compounds with multiple applications.

## 1. Introduction

*Argylia radiata* (L.) D. Don. is a herbaceous perennial plant that is part of the Bignoniaceae family. This species is native to Chile, dwelling in the Atacama Desert as a representative specie of the Flowering Desert, a random phenomenon caused by winter rains associated with the Child Current [1]. The *Argylia* plant sprouts and flourishes only when enough water is available. In the remaining time, they leave a massive tuberous root growing under the sandy soil, which can reach up to 3 kg of weight and 40 cm long. The *Argylia* plants only sprout when they have proper conditions, and they are able to wait several years before having the ideal water requirements.

Due to recent consumers and government concerns about global climate changes and the hydric crisis, this polyextremophile species has the potential to become a new class of low water requirement ornamental plant [2]. Moreover, other potential applications have not been explored yet. For this purpose, it is relevant to carry a plant phytochemical analysis to determine biologically active compounds.

Previous studies showed that the tribes *Bignonieae*, *Tecomeae* and *Eccremocarpeae* from the *Bignoniaceae* family have various bioactive compounds from the iridoid family [3]. In the case of *A. radiata*, 13 types of iridoids have been isolated: e.g., argylioside, catalpol, plantarenaloside, 8-epi-7-deoxyloganic acid, 7-deoxygardoside; geniposidic acid, mussaenosidic acid and radiatosides A, B, C, D, E and F [4,5,6,7,8,9]. For catapol iridoid glycoside, anticancer, neuroprotective and anti-inflammatory properties have been reported [10,11].

Our group recently studied polyphenolic distribution on *A. radiata* organs (flowers of different colors, leaves and roots). For the first time, ten polyphenolic compounds with antioxidant activity were reported [12]. Rutin was the principal compound identified in all organs, followed by quercetin and coumaric acid. Polyphenols are a broad family of compounds with a large spectrum of biological activities, including antimicrobials, antioxidants, anticancer and other beneficial effects on human health among others [13,14,15]. Polyphenols have applications in the food, cosmetic and pharma industries [16]. However, the small and sporadic availability of *A. radiata* plant biomass, dismissed further attempts to isolate both families of compounds at a massive scale.

To overcome the problem above, a novel tissue culture methodology was developed [17]. It allowed us to obtain enough and constant supply of *Argylia radiata* vitroplants for future ornamental, biotech or food use. This technique is an easy and efficient way to get an unlimited amount of biomass at a laboratory scale in a few weeks. It is known that, at certain growing stages, the future plants can form callus tissue prior to root and shoot formation [18]. After plant hormone treatments, these calluses can differentiate in organs, and vitroplants are obtained with their parents’ same genetic information. Thus, the obtained vitroplants are useful material for both biological testing and a source of novel phytochemicals [19].

The present work compares *Argylia radiata* plant roots and vitroplant organs (aerial part and callus) regarding their polyphenolic and flavonoid content. For this purpose, the effect of polar solvents (water, methanol and ethanol) for extract preparation is studied for the first time. UPLC-MS/MS determines the chemical composition of each plant extract. Moreover, the antioxidant capacity of the extract is determined with the ORAC-FL method, and the scavenging of free radical DPPH∙ and OH∙ is determined using the Electronic Spin Resonance (ESR) technique. Finally, the biological activity of the extracts is assessed by the cellular antioxidant activity.

## 2. Results and Discussion

### 2.1. Extraction from Tissue Culture Plant

**Figure 1:**
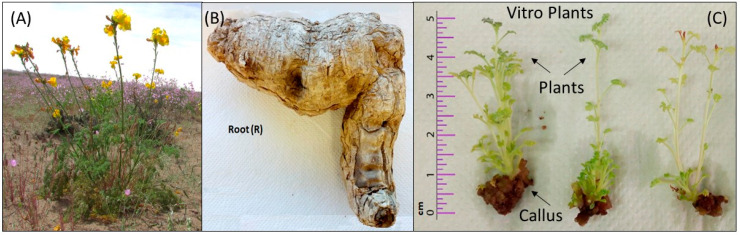
Atacama Desert wild *Argylia radiata* plant (**A**), its tuberous root (**B**) and vitroplants after five weeks of growth in MS media supplemented with 0.5 mg L^−1^ of BAP to promote callus formation (**C**).

Plant tissue culture is an excellent alternative to produce secondary metabolites with commercial uses [20] and to evaluate biological activities for novel phytochemical identification. In the case of A. radiata, different parts of the vitroplants, such as the callus (C) and aerial plant organs (stem + leaves) (P) were analyzed, and, for in vivo plants, only the tuberous root (R) was studied (Figure 1). For higher isolation of phenolic compounds, the plant root and vitro plant sample extractions were carried out with polar solvents (methanol (M), ethanol (E) and water (W). 

Thus, in the present work, we obtained a combination of solvent-plant extract coded as ethanol-callus (EC), ethanol-root (ER), ethanol-plant (EP), methanol-callus (MC), methanol-root (MR), methanol-plant (MP), water-callus (WC), water-root (WR) and water-plant (WP).

### 2.2. Phenolic Compounds Analysis

**Figure 2:**
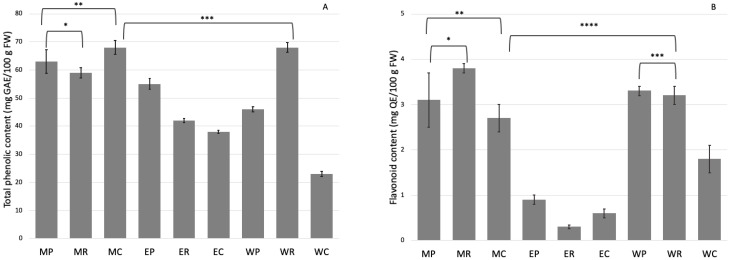
The total polyphenolic content (**A**) and flavonoid content (**B**) determined in *A. radiata* samples. Asterisk (*) indicates no significant differences between samples (*p* > 0.05; one-way ANOVA with a post hoc Tukey test). MP: plant methanolic extract; MR: root methanolic extract; MC: callus methanolic extract; EP: plant ethanolic extract; ER: root ethanolic extract; EC: callus ethanolic extract; WP: plant water extract; WR: root water extract; and WC: callus water extract.

The presence of polyphenols in plants is determined by genetic and environmental factors. They can interact directly with other cellular components, regulating cell processes as mitosis, cell elongation, senescence and cellular die [21]. In addition, they play an essential role in plant development under stress conditions due to its inherent antioxidant capacity. In the present work, different extraction solvents are used to isolate phenolic compounds. Comparative determination of the total polyphenolics content (TPC) and flavonoid content (FC) in plant root and vitroplants was performed. The results of TPC and FC quantification are shown in Figure 2.

It was determined that the highest amount of polyphenolic compounds was found in the WR and MC (69.3 and 68.1 mg GAE/100 g FW) extracts, respectively. The TPC of those extracts was significantly higher (*p* > 0.05) than determined for the remaining *Argylia* extracts. Likewise, it was observed that, depending on the extracting solvent, the TPC of C, P and R will vary while always maintaining a high content of TPC for methanolic extracts and the plant root starting material. 

It is known that methanol is a good solvent for polyphenol extraction. In a recent study carried out in *Argylia* plant organs [12], we determine that the highest TPC was found in the plant leaves (1520 mg GAE/100 g FW), followed by the flowers and roots (70 mg GAE/100 g FW). It is worth mentioning that those extracts were made using methanol as a solvent. The TPC content obtained for MR and WR extracts from the present work were not significantly different among them and they were slightly lower than MC and WR samples, which is interesting. 

General speaking, the TPC values from vitroplants are far from those observed in *Argylia* plant leaves and flowers collected in their natural habitat. Such differences can be due to the high stress at which those parts are exposed in nature, provoking secondary plant metabolism activations as one of their adaptive responses.

Larrazabal-Fuentes et al. [22] studied the extract of the *Acantholippia deserticola* (rica rica) plant, native to northern Chile. They determined a TPC value of 25 mg GAE/mL extract. A relationship with the TPC of *Argylia* was observed since these two plants share the same ecosystem.

In addition, Wong-Paz et al. [23] studied phenolic extraction from Mexican desert plants using an ethanolic mixture. They found TPC ranging from 70–332 mg GAE/100 g FW of *Jatropha dioica*, 52–310 mg GAE/100 g FW of *Flourensia cernua*, 112–350 mg GAE/100 g FW of *Turnera diffusa* and 390–1339 mg GAE/100 g FW of *Eucalyptus camaldulensis*. Results obtained for all *Argylia* methanolic extracts and WR extract were close to the TPC range from the first two plants, but only the EP treatment was close to *Jatropha dioica* extract polyphenolic content. However, the *Argylia* extract TPCs were 2- to 32-times lower than those determined in the other species. 

This result is interesting because the TPC found in several of our extracts is similar to plants grown under desert high-stress conditions (UV, temperature, salinity etc.). Then, the micropropagation technique can be considered an interesting tool for producing polyphenolic extracts from scarce and slow-growing desert plants. Plant polyphenols have been suggested as a promising source of antimicrobial agents, either alone or combined with commercial antibiotics, to develop new antibiotic therapies [24]. 

In addition, the beneficial effects of polyphenol consumption on chronic health conditions, cardiovascular disease, type 2 diabetes, neurological impairment and certain risk factors have been reported [25,26]. The flavonoid content indicates that methanol and water, but not ethanol, are good solvents for flavonoid extraction. This could be due to the polarity of the solvents and the affinity of the flavonoids with them. Researchers observed a significantly (*p* > 0.05) higher FC in the MR sample (3.94 mg QE/100 g FW), followed by the WP, WR, MP and MC samples, in the same order. However, non-significant differences were found among all those extracts. The ethanolic extracts contained low amounts of flavonoids (<1 mg QE/100 g FW), and the ER sample was the one with the lowest significant (*p* > 0.05) FC values. 

In [12], Morales-Tapia et al. studied the FC of *A. radiata* plants growing in their harsh natural habitat and observed that the FC content was higher in red flowers (43.5–187.6 mg QE/100 g FW) and leaves (126.6 mg QE/100 g FW) but lower in root pulp and cork (5.7 and 1.1 and mg QE/100 g FW). Those results agree with the present work, considering that the whole root sample was used for extract preparation. It is interesting to note that MP and WP vitro plant samples showed a similar concentration of flavonoids to the *A. radiata* tuberous root.

In addition, Sharma and Vig [27] determined the FC in different extracts of leaves from *Parkinsonia aculeata*, another desert plant. They found 1.3 mg R (rutin) E/100 g FW in methanolic extract and 0.6 mg RE/100 g FW water extract. In *Ephedra chilensis,* 2.3 mg QE/100 g DW was obtained in hexane, 9.5 mg QE/100 g DS in CH_2_Cl_2_ and 0.5 mg QE/100 g DS in ETOH [28]. Considering the above results, it is confirmed that the *A. radiata* plant has a high content of flavonoids compared to other plants that coexist in a similar ecosystem, which could influence the antioxidant capacity.

### 2.3. UPLC-MS/MS Analysis

**Figure 3:**
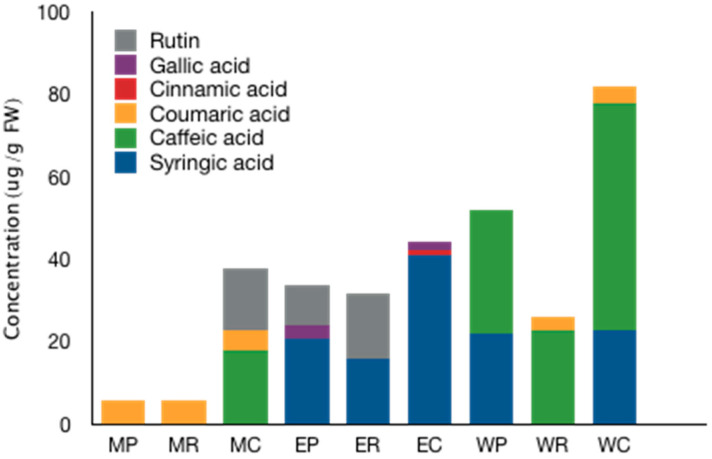
The chemical composition profile of *Argylia radiata* extracts determined by UPLC-MS/MS.

A chromatographic analysis was performed to determine the chemical composition of *Argylia* extracts obtained with different solvents. According to the results presented in Figure 3, a total of six polyphenols were identified among the samples. Syringic acid was the most frequent and abundant polyphenol in most extracts, followed by caffeic and coumaric acid, respectively. It is interesting to note that gallic acid was only determined in EP and EC samples, whereas MP and MR samples mostly contain coumaric acids. Syringic acid was present in roots, plants and callus tissue. 

Its presence could indicate a potential application of this extract since this polyphenol has shown antidiabetic activity [29], and antimicrobial activity inhibiting the growth of *Escherichia coli* was also reported [30]. Not present in all extracts but with high importance due to their biological activities is the polyphenol rutin. This flavonol glycoside has shown anti-inflammatory, antimicrobial and potent antioxidant activity as a scavenger of free radicals [31]. It is interesting to note that it was the most frequent metabolite found in wild *A. radiata* plants [12].

### 2.4. Antioxidant Capacity Studies

The antioxidant capacity of *Argylia* extracts was evaluated towards the biologically relevant ROS: peroxyl (ROO∙), hydroxyl (OH∙) radicals. Three different assays were selected, including oxygen radical absorbance capacity-fluorescein (ORAC-FL), spin trapping assay and cellular antioxidant activity (CAA) to achieve the goals. Trolox ((±)-6-hydroxy-2,5,7,8-tetramethylchroman-2-carboxylic acid), a vitamin E hydro-soluble derivative, was used as the standard. All measurements were performed in triplicate, and the values are expressed as the mean ± SD.

#### 2.4.1. Evaluation of ORAC-FL

The antioxidant capacity of *A. radiata* extracts was determined through the ORAC-FL method. This technique generates peroxyl and/or alkoxy radicals by thermolysis of 2,2′-azobis (2-methylpropionamidine) dihydrochloride (AAPH) at 37 °C [32,33]. These radicals oxidize the fluorescein probe, which implies a decrease in the fluorescence intensity of the probe as a function of time. Antioxidant compounds can delay oxidation of the probe through the transfer of hydrogen atoms [34,35,36].

As the reaction rate between generated radicals and fluorescein is low, the antioxidant capacity studied by this methodology is related to the reaction’s stoichiometry. However, the latter becomes relevant for pure compounds and not for complex mixtures of polyphenols, where it would only indicate the ability of an extract to scavenging oxygen-centered radicals [37].

Table 1 shows the antioxidant capacity for all extracts. The aqueous extracts showed the lowest ORAC antioxidant capacity. This may be due because only polyphenolic glycoside compounds and low molecular weight hydroxylated organic acids were extracted in this solvent. For instance, the caffeic acid present in all the aqueous extracts has an ORAC-FL index 3.47-times higher than the reference antioxidant Trolox [36]. These results would indicate that, in aqueous extracts, the compound’s combination would exert an antagonistic effect in relation to the antioxidant capacity analyzed by this methodology.

On the other hand, ethanolic and methanolic extracts presented a lower content of polyphenolic compounds in relation to aqueous extracts; where a high content of syringic acid was identified in ethanolic extract with a lower ORAC-FL index than caffeic acid [38]. This difference may be due to the presence of other polyphenolic compounds, such as rutin and gallic acid. However, the observed differences in the phenolic composition between methanolic and ethanolic extracts do not explain the similar antioxidant capacity values studied by this methodology. The latter can be explained by additive, synergistic or antagonistic effects between the compounds present in the extracts.

#### 2.4.2. Evaluation of OH· Scavenging by ESR

The spin trapping technique (ESR) evaluated hydroxyl elimination capacity. This technique consists of a reaction between a free radical with a diamagnetic molecule (spin trap), which generates a radical species with a longer half-life (spin adduct), which can be analyzed by electron spin resonance (ESR).

**Figure 4:**
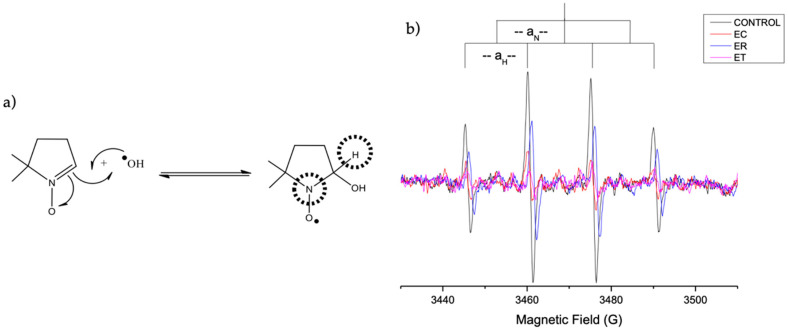
DMPO-OH spin adduct formation reaction (**a**) and EPR spectra of *A. radiata* ethanolic extracts (**b**).

Hydroxyl radicals (half-life of 10^−9^ s) [39] were generated by a non-catalytic Fenton reaction, and DMPO (5,5-dimethyl-1-pyrroline-N-oxide) was used as a spin trap. This allows the formation of radicals in basic medium without the use of Fe^2+^ as a catalyst. Figure 4b shows the ESR spectra (as an example, only the spectra obtained for the ethanolic extracts are shown), with four lines corresponding to the interaction between the unpaired electron with the nuclei of the nitrogen and hydrogen atoms observed. The hyperfine coupling constants (DMPO-OH, aN = aH ~14.76 G) obtained indicate the hydroxyl radical’s trapping [39].

The extracts of *Argylia* vitroplant in the three solvents used decreased the intensity of the DMPO-OH spin adduct signal, which implies a decrease in the OH· radical concentration. The radical scavenging values (Table 1) indicate that the EP extract showed a higher hydroxyl radical scavenging capacity, followed by the MP extract. The antioxidant capacity studied by this methodology was correlated with that determined by ORAC-FL, since both methods involve the transfer of the hydrogen atom. Where the methanolic and ethanolic P extracts presented the highest hydroxyl radical removal capacity.

#### 2.4.3. Evaluation of DPPH∙ Radical-Scavenging Activity by the EPR Method

A rapid, simple and economical method to determine antioxidant capacity involves the use of the free radical DPPH∙. The test is used to detect the antioxidant capacity of fruits, vegetables, juices and extracts. DPPH assays are generally classified by a sequential mechanism of electron transfer with proton loss, in which the DPPH radical accepts an electron followed by proton transfer from antioxidant compounds. 

Other authors postulated that a marginal mechanism of HAT (hydrogen atom transfer) may occur depending on the reaction medium and physicochemical properties of the compounds under study. The chemical determinations are usually made through a decrease of signal intensity in the spectrophotometry absorption spectrum. However, there could be several interferences with such results, such as the solvent used and the presence of undesired colored compounds in the samples [40].

**Figure 5:**
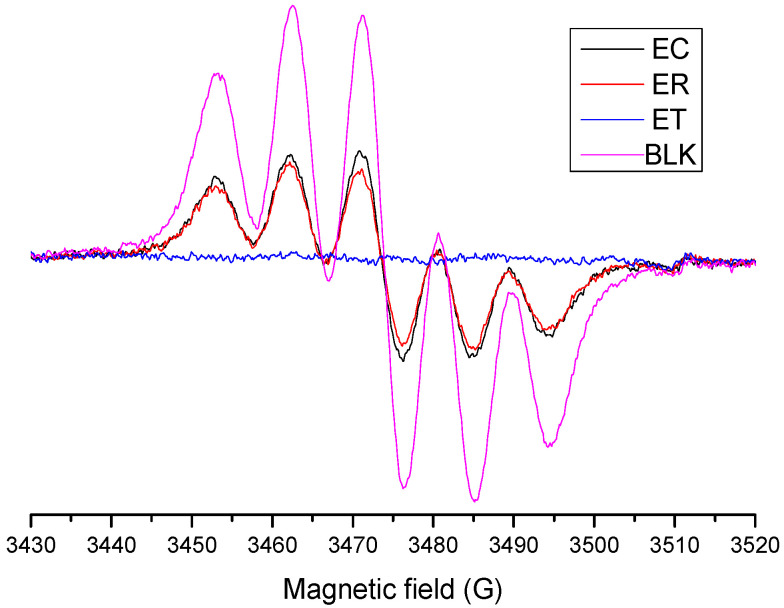
DPPH radical quenching of *Argylia* ethanolic extracts obtained by EPR analysis.

In this work, to determine a decrease in the concentration of radical without interference, the antioxidant capacity determination was made using the EPR technique. The EPR spectrum of DPPH∙ shows a six-line pattern in which the depletion of signal intensity is proportional to the antioxidant capacity of compounds present in the extracts. In Figure 5, as an example, only the spectra obtained for the ethanolic extracts are shown. The time to reach the endpoint of DPPH∙-EPR test depends on the medium and the properties of the molecules under study. 

The test results are expressed as a function of the DPPH∙ decrease rate (dynamic approach) and characterize the reactivity of the compounds present in the extracts. Unlike expressing the amount of DPPH∙ removed, which indicates the stoichiometry of DPPH∙ reaction with donor H (the amount of active OH groups) [40,41,42,43]. The results of the DPPH∙ assay are presented in Table 1. All extracts showed radical removal capacity. The ethanolic and methanolic extracts exhibited the highest capacity to eliminate the radical DPPH∙, with the extracts EP and MP as the ones with higher antioxidant capacities. The ability to eliminate the radical was the lowest for the aqueous extracts since DPPH has difficulty dissolving in water.

Larrazabal-Fuentes et al. [22] studied the antioxidant capacity of *Acantholippia deserticola* native to the Chilean desert, determining the capacity to eliminate the DPPH radical in a 50% ethanolic extract in water was 28.2 at 10 µg/mL. Those results corroborate our findings that the water content in the extracts influences the antioxidant capacity of the compounds when measured by the DPPH methodology. Therefore, it is better to work with methanolic or ethanolic extracts.

The UPLC-MS/MS results indicated that EP extract showed the highest DPPH radical scavenging capacity since it contains antioxidant polyphenols, including rutin, gallic acid and syringic acid. In [44], Motlhanka et al. studied the antioxidant activity of several pure compounds, such as rutin. They determined that the rutin DPPH scavenging capacity was approximately 60% at 25 ppm. Gow-Chin, Y. et al., [45] studied the antioxidant activity of gallic acid against the radical DPPH, determining that the activity was 43.9% at 4.7 mM. In addition, the antioxidant activity of syringic acid was approximately 60% at 50 µM (Zhou, K. et al.) [46]. 

With those above, it can be concluded that there is a synergy between the compounds in the EP extract, since its antioxidant capacity value against the DPPH radical is 97.6%. For the MP extract, a synergy study cannot be done as it has only coumaric acid. It should be noted that the technique to determine the quenching of DPPH radicals by EPR is straightforward, since it makes a direct measurement of the radical in the presence of the antioxidant, while techniques, such as ORAC, DPPH, etc., are indirect measurement techniques because the results are obtained according to the variation of probes etc.

### 2.5. Evaluation of Cellular Antioxidant Activity (CAA)

The compound DCFH_2_-DA has been extensively used as a probe for the detection of oxidative stress that can reflect the overall oxidative status of the cell. The dye, DCFH_2_-DA, diffuses passively through the cellular membrane and is transformed into DCFH_2_—a non-fluorescent compound that emits fluorescence when it is oxidized to 2,7-dichlorofluorescein (DCF) (λ_excitation_ = 498 nm; λ_emission_ = 522 nm) by intracellular ROS—due to intracellular esterase activity. The AAPH is usually employed as the generator of peroxyl radicals. Therefore, it is generally accepted that hydrophilic antioxidants can interact more appropriately with the radicals generated by AAPH than hydrophobic antioxidants.

In Table 2, results from the cellular antioxidant activity of *Argylia* vitroplant extracts and root extracts are presented. It is worth mentioning that two concentrations of 1 and 10 µg/mL were evaluated, hoping that the antioxidant capacity increases as the concentration increases. In turn, if the concentration of 10 µg/mL is increased, the sensitivity of the method decreases. The extracts showed cellular antioxidant activity related to the type of solvent used in the extraction of antioxidant polyphenolic compounds and the vitro plant part used. They also have antioxidant activity in a dose-dependent manner, increasing along with the extract concentration assayed.

In Table 2, the results of the cellular antioxidant activity of *Argylia* vitroplant extracts and root extracts are presented. It is worth mentioning that two concentrations of 1 and 10 µg/mL were evaluated in order to compare these results with those described in the literature. It was found that the extracts of P (stem and leaves) presented the highest cellular antioxidant activity in the three solvents used, as with the results obtained through ORAC-FL. The cellular antioxidant activity determined was higher than that obtained recently by Zúñiga-López et al. [47] for ethanolic extracts of *Salvia hispanica* L. (chia) leaves, which obtained a percentage CAA of 28 ± 2 and 13.2 ± 0.6 (evaluated at 10 µg/mL) for the white and black phenotypes, respectively. Those results points to *Argylia* vitroplant extracts as promising antioxidants for biotechnological applications.

On the other hand, the methanolic extracts of *Argylia* vitroplant showed the highest cellular antioxidant activity. The latter suggests that the polyphenolic compounds extracted in this solvent were more bioavailable for RAW 264.7 cells than the compounds extracted in ethanol and water. This outcome could also be attributed to the higher polyphenolic content found in the methanolic extracts or to their chemical composition. Further analysis to clarify this point is under progress in our group.

In particular, the MP and MR extracts showed the highest cellular antioxidant activity at both concentrations. They displayed from 40–60-times higher activity at 1 µg/mL than the other ones. These results indicate that compounds extracted with methanol showed the highest effect as cellular antioxidants. On the other side, the antioxidant activity of ethanol and water extracts at 1 µg/mL concentration were similar, ranging from 17.6% to 23.4%. At 10 µg/mL, the antioxidant activity tendency is similar to at lower concentrations, except for the vitroplant water extract, which showed a high cellular antioxidant activity.

Polyphenolic extracts showing cellular antioxidant activity indicate that phenolic compounds can diffuse into the cell membrane and exert their activity into the cytoplasm. It is known that this antioxidant method accounts for aspects of uptake, metabolism and the location of antioxidant compounds within cells [48]. That makes it relevant and useful for natural product bioactivity evaluation. 

Wolfe et al. [49] studied the cellular antioxidant capacity of pure compounds and different fruit extracts. These authors determined that quercetin was the best cellular antioxidant with 100% antioxidant activity, while caffeic acid and catechin have the lowest training of approximately 3% and 2%, respectively. Therefore, when evaluating the MP and MR extracts containing only coumaric acid, it could be concluded that coumaric acid has a better cellular antioxidant capacity when compared with catechin and caffeic acid but a lower antioxidant capacity compared with quercetin. 

Likewise, in all the fruit extracts evaluated, the blueberry extract had the highest antioxidant capacity 8.70 µM quercetin equivalent (QE)/100 µmol of total phenols. In contrast, the green grape extract had the lowest activity 1.04 µM of QE/100 µmol of total phenols. Therefore, when comparing with the antioxidant capacity obtained by the MP and MR extracts, they observed that the extracts were good antioxidants compared to the fruit extracts.

All those results confirm *Argylia* vitroplant extracts’ potential application as antioxidant compounds for different in vivo applications. In addition, these results demonstrate the usefulness of the tissue culture technique to obtain plant biomass at a large scale. Moreover, its relevance as a tool for obtaining interesting content of phytochemicals from extremophiles and scarcely available plant biomass in short times are highlighted.

## 3. Materials and Methods

### 3.1. Obtaining of Plant Material

In 2017, herbaceous shoots and tuberous storage roots of *Argylia radiata* were collected from Bahía Inglesa, Caldera, Región de Atacama, Chile (27°07′32″ S; 70°48′53″ W). The sprouts were protected with wet paper towels inside plastic bags during their transport, and they were kept at 4 to 7 °C until they were initiated while the roots were put on mesh and stored at room temperature.

The in vitro lines were obtained according to the described procedure by Morales [17]. The shoots were sterilized by dipping in a fungicide solution (Captan 20 g/L) for 30 min. After that, they were passed through an ethanol solution at 70% (*v*/*v*) and washed in a solution of commercial bleach at 20% (*v*/*v*) for 20 min. After, the material was rinsed three times with sterile water. Finally, nodal sections were planted in Murashige & Skoog medium [50], supplemented with 30 g/L of sucrose, 6.5 g/L of agar. Explants were kept in a growth chamber at 23 to 25 °C and 16 light hours. After six weeks, the new sprouts were isolated to a fresh medium. To obtain callus tissue, vitroplants of *Argylia* were cultivated for 5 weeks in growth media enriched with 0.5 mg/L of 6-Benzylaminopurine (BAP).

### 3.2. Plant Extract Preparation

The A. radiata vitroplants obtained (Figure 1) were separated in callus (C) and plant (P) for polyphenol analysis. At the same time, *Argylia* tuberous roots, collected in the Atacama Desert, were obtained. Samples (1 g) were used to extract preparation with four different solvents: methanol (M), ethanol (E) and water (W). The solid:liquid ratio used in all cases was 1:10, and the extractions were performed at room temperature for 3 h under orbital shaking. Then, the extracts were centrifuged, and the liquid phases were stored at −20 °C for further analysis.

### 3.3. Total Phenolic Determination

The total phenolics content (TP) was determined according to the Folin Ciocalteu (FC) method. A 500 µL aliquot was mixed with 2.5 mL of the FC reagent 1:10 (*v*/*v*) and 2 mL of a solution of Na_2_CO_3_ (75 g/L) and incubated 60 min at room temperature. The absorbance of the resulting blue solution was measured at 760 nm using an Agilent 8453 UV-visible spectrophotometer (Palo Alto, CA, USA). The calibration curve was performed using gallic acid, and the results are expressed as milligrams of gallic acid (GA) equivalents per gram.

### 3.4. Flavonoids Determination

The flavonoid content was estimated according to the aluminum chloride method [51]. A total of 500 µL of the extract was mixed with 500 µL of 2% AlCl_3_ in methanol and incubated 60 min at room temperature. The absorbance was measured at 420 nm using an Agilent 8453 UV-visible spectrophotometer (Palo Alto). The total flavonoid contents were calculated as milligrams of quercetin equivalents per gram from a calibration curve.

### 3.5. UPLC-MS/MS Determination

An ABSciex triple Quad 4500 mass spectrometer equipped with an electrospray (TurboV) interface coupled to an Eksigent Ekspert Ultra LC100 with Ekspert Ultra LC100- XL autosampler system (AB/Sciex Concord, Concord, ON, Canada) was used for the determination of selected polyphenols. The chromatographic separation was carried out using a gradient elution of 0.1% formic acid in water (A) and methanol (B) as the mobile phase. The gradient was programmed as follows: 0–1 min, 15% B; 1–17 min, 15–100% B; 17–21 410 min 100–100% B; 21–22 min, 100–15% B; and 22–25 min, 15–15% B. 

The injection volume was 10 μL, and the flow rate was kept at 0.5 mL/min. A LiChrospher 100 RP-18 end-capped column (125 mm × 4 mm id, 5 μm) (Merck, Darmstadt, Germany) was used with a controlled temperature of 40 °C. Mass spectrometry analysis was operated in negative mode; the declustering potential (DP) and collision energy (CE) were optimized for each analyte. Compounds were identified by comparing MS/MS data for those obtained with commercially available standards according to Table 3. Quantification was performed with calibration curves ranging from 10 to 500 µg L^−1^.

### 3.6. Antioxidant Assays

#### 3.6.1. Oxygen Radical Antioxidant Capacity-Fluorescein (ORAC-FL)

The analyses were carried out in an EnSpire multi-detection microplate reader from PerkinElmer, using white polystyrene 96-well plates, purchased from Nunc (Roskilde, Denmark). The fluorescence was read from the top with an excitation wavelength of 485/20 nm and an emission filter of 528/20 nm.

The reaction was carried out in 75 mM sodium phosphate buffer (pH 7.4), in a 200 μL final volume. Fluorescein (FL, 40 nM, final concentration) and extract solutions in phosphate buffer (pH 7.4) were placed in each well of a 96-well plate. The mixtures were pre-incubated at 37 °C for 15 min, and 2,2’-azo-bis(2-amidinopropane)dihydrochloride (AAPH) solution (18 mM, final concentration) was added [35]. The microplate was immediately placed in the reader and automatically shaken prior to each reading. The fluorescence was recorded every 1 min for 80 min. A blank with FL and AAPH using buffer instead of the extract solution was used in each assay. 

Five calibration solutions of Trolox (2–20 µM) as the antioxidant were also used in each assay. The inhibition capacity was expressed as concentration Trolox equivalents (TE) per 100 g of dry mass, and this was quantified by integration of the area under the fluorescence decay curve (AUC). All reaction mixtures were prepared in triplicate, and at least three independent assays were performed for each sample [36,52].

#### 3.6.2. Evaluation of OH· Scavenging by ESR

The ESR (electron spin resonance) spectra were recorded in the X band (9.7 GHz) using a Bruker ECS 106 spectrometer with a rectangular cavity and 50-kHz field modulation, which was equipped with a high-sensitivity resonator at room temperature. The spectrometer conditions were: microwave frequency 9.81 GHz; microwave power 20 mW; modulation amplitude 0.91 G; receiver gain 59 db; time constant 81.92 ms; and conversion time 40.96 ms. 

The scavenging activity of each derivative was estimated by comparing the DMPO-OH adduct signals in the antioxidant-radical reaction mixture and the control reaction at the identical reaction time. The scavenging activity is expressed as the scavenging percentage of hydroxyl radicals. The compound reactivity against hydroxyl radicals was investigated using the non-catalytic Fenton type method. 

The samples were prepared as follows: 100 μL of the solvent used in extraction and 50 μL of NaOH (25 mM) were mixed. Then, 50 μL of DMPO (5,5-dimethyl-1-pyrroline-N-oxide, 30 mM final concentration), 50 μL of the sample (20 mM in solvent used in extraction) and 50 μL of hydrogen peroxide (30%) were sequentially added. The mixture was put into an ESR cell, and the spectrum was recorded after five minutes of reaction [36,52,53].

#### 3.6.3. DPPH Radical Scavenging Activity Evaluated by the ESR Method

The 1,1-diphenyl-2-picrylhydrazyl (DPPH∙) radical scavenging capacities of the extracts were determined by electron spin resonance (ESR) spectrometry. All the reaction mixtures contained 1.0 mM of DPPH∙ (volume, 50 µL; final concentration, 250 mM) and 150 µL of each extract under study. Control experiments (without extract) were carried out. ESR signals were recorded after 3 min of reaction, as this was the time required to reach the reaction equilibrium. All the spectra were recorded in three scans in the same period. The scavenging activity of each compound was estimated by comparing the DPPH signals in the antioxidant–radical reaction mixture and the control reaction, at the same reaction time [40]. The results were expressed as the DPPH scavenging percentage.

#### 3.6.4. Evaluation of the Antioxidant Activity in RAW 264.7 Cells

The cellular antioxidant activity (CAA) was evaluated in RAW 264.7 cells using 2′,7′-dichlorodihydrofluorescein diacetate (DCFH2-DA) as a fluorescent probe. The cells were seeded in 96-well flat bottom microplates of sterile white polystyrene (Nunc, Denmark) at a concentration of 50,000 cells per well and incubated for 24 h at 37 °C and 5% CO_2_ in RPMI 1640 culture medium. The cells were washed with 150 µL of phosphate buffered saline (PBS) (pH = 7.4) and incubated for 1 h with 100 µL of RPMI 1640 containing 25 mM DCFH2-DA. 

The extracts under study were added at final concentrations of 1 and 10 ppm. After 1 h of incubation, the medium was discarded, and the cells were washed twice gently with 200 µL of PBS. They were then incubated with AAPH at a final concentration of 600 mM (in PBS). The fluorescence was measured immediately after the addition of AAPH in a Perkin–Elmer EnSpire in 96-well plates at 37 °C using an excitation wavelength of 485 nm and an emission wavelength of 538 nm [36,40,48]. The evaluation was performed every minute for 1 h, and the CAA values were calculated using equation (x):

$$\mathrm{CAA}\%=\left(\frac{\Delta F-\Delta F_{\mathrm{AH}}}{\Delta F}\right)\times 100$$

F = fluorescence intensity in the presence of free radicals, without extract. F_AH_ = fluorescence intensity in the presence of free radicals and extract. All measurements were made in the same period.

## 4. Conclusions

*Argylia radiata* is a polyextremophyle plant from the Atacama Desert that does not have normal seasonal growth. That is why tissue culture was used as a tool for high biomass production out of season. It could be used as a tool to investigate physiological changes that occur in plant cells (callus) and organs (aerial part) and, at the same time, as a source of value-add biotechnological compounds. In this work, extraction with polar solvents allowed for the isolation of polyphenolic and flavonoid compounds. Methanol appeared to be the best solvent to isolate polyphenolic compounds (60–67 mg GAE/100 g fresh tissue) from all tested tissue. 

In contrast, water was the best solvent for isolating such compounds from natural plant roots (65 mg GAE/100 g fresh tissue). Both solvents showed a similar capacity for isolating flavonoids, and the callus tissue contained the lowest amount of such compounds. HPLC-MS/MS identified eight different secondary metabolites in the studied tissues. Caffeic acid, syringic acid and rutin were found in higher quantities. The chemical and biological antioxidant capacity establishes that the *Argylia* plant extracts show antioxidant properties against different radical species. 

Moreover, the extract compounds can cross the plant cell wall barrier and displayed an antioxidant capacity in a dose-dependent manner inside cells. All these results suggest the potential application of vitroplant extracts as antioxidant compounds for different applications, i.e., food packaging, cosmetics, nutraceuticals, biopharma and other biotechnological uses. Finally, this work will open an opportunity for using extremophilic plants from the world’s driest desert as a source of valuable compounds.

## Figures and Tables

**Table 1:** Results from the antioxidant capacity of *Argylia* vitroplant and root extracts measured by fluorescence and ESR methods.

Samples	ORAC-FL (µM TE/g FW)	Scavenging OH∙ (%) *	Scavenging DPPH∙ (%) *
MP	1031.3 ± 4.2	81.7 ± 1.1	87.6 ± 2.0
MR	982.3 ± 2.5	73.5 ± 2.1	76.7 ± 1.9
MC	917.3 ± 3.1	67.4 ± 1.3	68.9 ± 1.2
EP	1103.4 ± 3.6	97.1 ± 1.1	97.6 ± 2.0
ER	837.8 ± 2.6	59.3 ± 1.2	56.7 ± 1.9
EC	989.3 ± 3.4	81.9 ± 1.2	61.9 ± 1.2
WP	869.1 ± 1.8	57.6 ± 2.1	27.6 ± 2.1
WR	798.5 ± 1.9	56.7 ± 2.1	16.7 ± 1.1 ^a^
WC	805.4 ± 2.1	58.9 ± 1.2	18.9 ± 1.0 ^a^

* Determined at 10 µg/mL extract.

**Table 2:** Cellular antioxidant activity (%) of *Argylia radiata* extracts is determined at different concentrations.

Samples	Cellular Antioxidant Activity (%)	
	1 (µg/mL)	10 (µg/mL)
MP	44.6 ± 3.2	51.0 ± 1.1
MR	33.8 ± 1.3	45.5 ± 1.3
MC	21.7 ± 2.0	37.4 ± 2.1
EP	23.4 ± 1.1	39.7 ± 1.8
ER	20.3 ±1.5	32.5 ± 1.6
EC	20.1 ± 1.3	33.1 ± 1.8
WP	21.6 ± 1.8	41.1 ± 1.3
WR	17.6 ± 1.4	34.3 ± 1.5
WC	20.4 ± 1.9	27.8 ± 1.3

**Table 3:** MS/MS data for the identification of compounds in *Argylia radiata* extracts.

Standard	Mass Q1	Mass Q3	DP	CE
Apigenin	268,958	117,000	−130,000	−130,000
Caffeic Acid	178,949	135,000	−70,000	−70,000
Catechin	289,038	245,000	−100,000	−100,000
Chlorogenic Acid	353,111	191,000	−75,000	−75,000
Chrysin	253,048	142,900	−120,000	−120,000
Cinnamic Acid	146,974	103,100	−55,000	−55,000
Cumaric Acid	162,913	119,000	−70,000	−70,000
Epicatechin	288,985	244,900	−110,000	−110,000
Ferulic Acid	192,974	146,900	−50,000	−50,000
Gallic Acid	168,922	124,900	−70,000	−70,000
3-Hydroxybenzoic Acid	136,958	92,900	−50,000	−50,000
Luteolin	285,022	133,000	−125,000	−125,000
Myricetin	316,934	150,900	−120,000	−120,000
Pinocembrin	255,062	212,900	−95,000	−95,000
Quercetin	301,010	150,900	−115,000	−115,000
Resveratrol	227,004	185,000	−100,000	−100,000
Rutin	609,007	299,800	−170,000	−170,000
Sinapic Acid	223,021	207,900	−75,000	−75,000
Syringic Acid	196,981	181,900	−65,000	−65,000
Vanillic Acid	166,844	122.900	−50,000	−50,000

## Data Availability

Not applicable.

## Sample Availability

Samples of the extracts are available from the authors.

## References

[1] Riedemann P., Aldunate G., Teillier S., Segunda (2016). Flora Nativa de Valor Ornamental; Identificación y Propagación, Chile Zona Norte.

[2] Morales-Tapia P., Gambardella M., Gómez M., Montenegro G. (2019). Morpho-anatomical adaptations of *Argylia radiata* (L.) D. Don to an arid environment. Flora Morphol. Distrib. Funct. Ecol. Plants.

[3] Lino Von Poser G., Schripsema J., Henriques A.T., Rosendal Jensen S. (2000). The distribution of iridoids in Bignoniaceae. Biochem. Syst. Ecol..

[4] Bianco A., Passacantilli P., Righi G., Nicoletti M., Serafini M., Garbarino J.A., Gambaro V. (1986). Argylioside, a dimeric iridoid glucoside from *Argylia radiata*. Phytochemistry.

[5] Bianco A., Passacantilli P., Rispoli C. (1986). Radiatoside, a new bisiridoid from *Argylia radiata*. J. Nat. Prod..

[6] Bianco A., Passacantilli P., Righi G., Nicoletti M., Serafini M., Garbarino J.A., Gambaro V., Chamy M.C. (1987). Radiatoside B and C, Two New Bisiridoid Glucosides from *Argylia radiata*. Planta Med..

[7] Bianco A., Passacantilli P., Garbarino J.A., Gambaro V., Serafini M., Nicoletti M., Rispoli C., Righi G. (1991). A New Non-Glycosidic Iridoid and a New Bisiridoid from *Argylia radiata*. Planta Med..

[8] Bianco A., Marini E., Nicoletti M., Foddai S., Garbarino J.A., Piovano M., Chamy M.T. (1992). Bis-iridoid glucosides from the roots of *Argylia radiata*. Phytochemistry.

[9] Bianco A., Bonadies F., Cianciolo V., Melchioni C., Ramunno A., Dezzi S., Nicoletti M., Serafini M., Ballero M. (2002). Monoterpene alkaloids from *Argylia radiata*. Nat. Prod. Lett..

[10] Zhang X., Jin C., Li Y., Guan S., Han F., Zhang S. (2013). Catalpol improves cholinergic function and reduces inflammatory cytokines in the senescent mice induced by d-galactose. Food Chem. Toxicol..

[11] Zou G.L., Zhong W.L., Wu F., Wang X.X., Liu L. (2019). Catalpol attenuates cardiomyocyte apoptosis in diabetic cardiomyopathy via Neat1/miR-140–5p/HDAC4 axis. Biochimie.

[12] Morales-Tapia P., Cabrera-Barjas G., Giordano A. (2020). Polyphenolic distribution in organs of *Argylia radiata*, an extremophile plant from Chilean Atacama desert. Nat. Prod. Res..

[13] Dzah C.S., Duan Y., Zhang H., Serwah Boateng N.A., Ma H. (2020). Latest developments in polyphenol recovery and purification from plant by-products: A review. Trends Food Sci. Technol..

[14] Dzah C.S., Duan Y., Zhang H., Wen C., Zhang J., Chen G., Ma H. (2020). The effects of ultrasound assisted extraction on yield, antioxidant, anticancer and antimicrobial activity of polyphenol extracts: A review. Food Biosci..

[15] Ma G., Chen Y. (2020). Polyphenol supplementation benefits human health via gut microbiota: A systematic review via meta-analysis. J. Funct. Foods.

[16] Tresserra-Rimbau A., Lamuela-Raventos R.M., Moreno J.J. (2018). Polyphenols, food and pharma. Curr. Knowl. Dir. Future Res. Biochem. Pharmacol..

[17] Morales P. (2019). *Argylia radiata* micropropagation, a biotechnological tool to domesticate a new ornamental crop. Acta Hortic..

[18] Driscoll K., Deshpande A., Chapp A., Li K., Datta R., Ramakrishna W. (2019). Anti-inflammatory and immune-modulating effects of rice callus suspension culture (RCSC) and bioactive fractions in an in vitro inflammatory bowel disease model. Phytomedicine.

[19] Wang J., Li J., Li J., Li J., Liu S., Huang L., Gao W. (2017). Production of Active Compounds in Medicinal Plants: From Plant Tissue Culture to Biosynthesis. Chin. Herb. Med..

[20] Paek K.Y., Murthy H.N., Zhong J.J. (2014). Production of Biomass and Bioactive Compounds Using Bioreactor Technology.

[21] Rodrigo R., Libuy M., Watson R.R. (2014). Modulation of Plant Endogenous Antioxidant Systems by Polyphenols. Polyphenols in Plants: Isolation, Purification and Extract Preparation.

[22] Larrazabal-Fuentes M., Palma J., Paredes A., Mercado A., Neira I., Lizama C., Sepulveda B., Bravo J. (2019). Chemical composition, antioxidant capacity, toxicity and antibacterial activity of the essential oils from *Acantholippia deserticola* (Phil.) Moldenke (Rica rica) and *Artemisia copa* Phil. (Copa copa) extracted by microwave-assisted hydrodistillation. Ind. Crops Prod..

[23] Wong-Paz J.E., Contreras-Esquivel J.C., Rodríguez-Herrera R., Carrillo-Inungaray M.L., López L.I., Nevárez-Moorillón G.V., Aguilar C.N. (2015). Total phenolic content, in vitro antioxidant activity and chemical composition of plant extracts from semiarid Mexican region. Asian Pac. J. Trop. Med..

[24] Álvarez-Martínez F.J., Barrajón-Catalán E., Encinar J.A., Rodríguez-Díaz J.C., Micol V. (2018). Antimicrobial Capacity of Plant Polyphenols against Gram-positive Bacteria: A Comprehensive Review. Curr. Med. Chem..

[25] Sun L., Miao M. (2020). Dietary polyphenols modulate starch digestion and glycaemic level: A review. Crit. Rev. Food Sci. Nutr..

[26] Cao H., Ou J., Chen L., Zhang Y., Szkudelski T., Delmas D., Daglia M., Xiao J. (2019). Dietary polyphenols and type 2 diabetes: Human Study and Clinical Trial. Crit. Rev. Food Sci. Nutr..

[27] Sharma S., Vig A.P. (2013). Evaluation of in vitro antioxidant properties of methanol and aqueous extracts of *Parkinsonia aculeata* L. leaves. Sci. World J..

[28] Mellado M., Soto M., Madrid A., Montenegro I., Jara-Gutiérrez C., Villena J., Werner E., Godoy P., Aguilar L.F. (2019). In vitro antioxidant and antiproliferative effect of the extracts of *Ephedra chilensis* K Presl aerial parts. BMC Complement. Altern. Med..

[29] Muthukumaran J., Srinivasan S., Venkatesan R.S., Ramachandran V., Muruganathan U. (2013). Syringic acid, a novel natural phenolic acid, normalizes hyperglycemia with special reference to glycoprotein components in experimental diabetic rats. J. Acute Dis..

[30] Campos F.M., Couto J.A., Hogg T.A. (2003). Influence of phenolic acids on growth and inactivation of Oenococcus oeni and *Lactobacillus hilgardii*. J. Appl. Microbiol..

[31] Chua L.S. (2013). A review on plant-based rutin extraction methods and its pharmacological activities. J. Ethnopharmacol..

[32] Dorta E., Fuentes-Lemus E., Aspée A., Atala E., Speisky H., Bridi R., Lissi E., López-Alarcón C. (2015). The ORAC (oxygen radical absorbance capacity) index does not reflect the capacity of antioxidants to trap peroxyl radicals. RSC Adv..

[33] Dorta E., Aspée A., Pino E., González L., Lissi E., López-Alarcón C. (2017). Controversial alkoxyl and peroxyl radical scavenging activity of the tryptophan metabolite 3-hydroxy-anthranilic acid. Biomed. Pharmacother..

[34] Ou B., Hampsch-Woodill M., Prior R.L. (2001). Development and Validation of an Improved Oxygen Radical Absorbance Capacity Assay Using Fluorescein as the Fluorescent Probe. J. Agric. Food Chem..

[35] Alarcón E., Campos A.M., Edwards A.M., Lissi E., López-Alarcón C. (2008). Antioxidant capacity of herbal infusions and tea extracts: A comparison of ORAC-fluorescein and ORAC-pyrogallol red methodologies. Food Chem..

[36] Pérez-Cruz K., Moncada-Basualto M., Morales-Valenzuela J., Barriga-González G., Navarrete-Encina P., Núñez-Vergara L., Squella J.A., Olea-Azar C. (2018). Synthesis and antioxidant study of new polyphenolic hybrid-coumarins. Arab. J. Chem..

[37] López-Alarcón C., Aspée A., Lissi E. (2012). Influence of the target molecule on the ORAC index. ACS Symp. Ser..

[38] Koroleva O., Torkova A., Nikolaev I., Khrameeva E., Fedorova T., Tsentalovich M., Amarowicz R. (2014). Evaluation of the antiradical properties of phenolic acids. Int. J. Mol. Sci..

[39] Villamena F.A. (2017). EPR Spin Trapping. Reactive Species Detection in Biology.

[40] Mura F., Silva T., Castro C., Borges F., Zuñiga M.C., Morales J., Olea-Azar C. (2014). New insights into the antioxidant activity of hydroxycinnamic and hydroxybenzoic systems: Spectroscopic, electrochemistry, and cellular studies. Free. Radic. Res..

[41] Guo Q., Zhao B., Shen S., Hou J., Hu J., Xin W. (1999). ESR study on the structure-antioxidant activity relationship of tea catechins and their epimers. Biochim. Biophys. Acta-Gen. Subj..

[42] Foti M.C., Daquino C., Geraci C. (2004). Electron-Transfer Reaction of Cinnamic Acids and Their Methyl Esters with the DPPH∙ Radical in Alcoholic Solutions. J. Org. Chem..

[43] Espinoza M., Olea-Azar C., Speisky H., Rodríguez J. (2009). Determination of reactions between free radicals and selected Chilean wines and transition metals by ESR and UV-vis technique. Spectrochim. Acta-Part A Mol. Biomol. Spectrosc..

[44] Mothanka D., Habtemariam S., Houghton P. (2008). Free radical scavenging activity of crude extracts and 4′-o-methylepigallocatechin isolated from roots of cassine transvaalensis burtt-davy from Botswana. Afr. J. Biomed. Res..

[45] Yen G.-C., Duh P.-D., Tsai H.-L. (2002). Antioxidant and pro-oxidant properties of ascorbic acid and gallic acid. Food Chem..

[46] Zhou K., Yin J.-J., Yu L. (2006). ESR determination of the reactions between selected phenolic acids and free radicals or transition metals. Food Chem..

[47] Zúñiga-López M.C., Maturana G., Campmajó G., Saurina J., Núñez O. (2021). Determination of Bioactive Compounds in Sequential Extracts of Chia Leaf (*Salvia hispanica* L.) Using UHPLC-HRMS (Q-Orbitrap) and a Global Evaluation of Antioxidant In Vitro Capacity. Antioxidants.

[48] Wolfe K.L., Rui H.L. (2007). Cellular antioxidant activity (CAA) assay for assessing antioxidants, foods, and dietary supplements. J. Agric. Food Chem..

[49] Wolfe K.L., Kang X., He X., Dong M., Zhang Q., Liu R.H. (2008). Cellular Antioxidant Activity of Common Fruits. J. Agric. Food Chem..

[50] Murashige T., Skoog F. (1962). A revised medium for rapid growth and bioassay with tobacco tissue cultures. Physiol. Plant..

[51] Woisky R.G., Salatino A. (1998). Analysis of propolis: Some parameters and procedures for chemical quality control. J. Apic. Res..

[52] Moncada-Basualto M., Lapier M., Maya J.D., Matsuhiro B., Olea-Azar C., Delogu G.L., Uriarte E., Santana L., Matose M.J. (2018). Evaluation of Trypanocidal and Antioxidant Activities of a Selected Series of 3-amidocoumarins. Med. Chem..

[53] Matos M.J., Mura F., Vazquez-Rodriguez S., Borges F., Santana L., Uriarte E., Olea-Azar C. (2015). Study of coumarin-resveratrol hybrids as potent antioxidant compounds. Molecules.

